# RTN1-C mediates cerebral ischemia/reperfusion injury via ER stress and mitochondria-associated apoptosis pathways

**DOI:** 10.1038/cddis.2017.465

**Published:** 2017-10-05

**Authors:** Lingli Gong, Yuewen Tang, Ran An, Muya Lin, Lijian Chen, Jian Du

**Affiliations:** 1 Department of Biochemistry and Molecular Biology, School of Basic Medical Sciences, Anhui Medical University, Hefei 230032, China; 2 Anhui Key Laboratory of Zoonoses, Anhui Medical University, Hefei 230032, China; 3 Anhui Provincial Laboratory of Pathogen Biology, Anhui Medical University, Hefei 230032, China; 4 Department of Anesthesiology, the First Affiliated Hospital of Anhui Medical University, Hefei, 230032, China

## Abstract

The reticulon family has been found to induce apoptosis, inhibit axon regeneration and regulate protein trafficking. However, little is known about the mechanisms of how reticulon proteins are involved in neuronal death-promoting processes during ischemia. Here, we report that the expression of Reticulon Protein 1-C (RTN1-C) was associated with the progression of cerebral ischemia/reperfusion (I/R) injury. Using a combination of rat middle cerebral artery occlusion (MCAO) stroke and oxygen-glucose deprivation followed by reoxygenation (OGD/R) models, we determined that the expression of RTN1-C was significantly increased during cerebral ischemic/reperfusion. RTN1-C overexpression induced apoptosis and increased the cell vulnerability to ischemic injury, whereas RTN1-C knockdown reversed ischemia-induced apoptosis and attenuated the vulnerability of OGD/R-treated neural cells. Mechanistically, we demonstrated that RTN1-C mediated OGD/R-induced apoptosis through ER stress and mitochondria-associated pathways. RTN1-C interacted with Bcl-xL and increased its localization in the ER, thus reducing the anti-apoptotic activity of Bcl-xL. Most importantly, knockdown of *Rtn1-c* expression *in vivo* attenuated apoptosis in MCAO rats and reduced the extent of I/R-induced brain injury, as assessed by infarct volume and neurological score. Collectively, these data support for the first time that RTN1-C may represent a novel candidate for therapies against cerebral ischemia/reperfusion injury.

Ischemic stroke remains as a major cause of disability and death in today’s world.^1, 2, 3^ The primary treatments for ischemic stroke are recanalization therapies, which are believed to replenish nutrients and oxygen, as well as remove toxic metabolites.^4, 5^ However, restoration of blood flow is sometimes connected with worsening of tissue injury and inflammatory response.^6^ The development of new treatments requires a comprehensive understanding of the diverse mechanisms that are responsible for neuronal death during ischemic brain damage. Cerebral ischemia and reperfusion (I/R) can activate various programs of cell death, such as necrosis, apoptosis or autophagy-associated cell death.^7, 8, 9^ Among these, apoptosis has been regarded as the key event for brain damage in cerebral ischemia.^9^

The activation of classical apoptosis occurs through two main pathways.^9, 10^ One of them is the extrinsic pathway, which is initiated through the activation of cell-surface death receptors such as Fas, and results in the activation of caspase-8 or -10. The other is the intrinsic pathway, also called the mitochondrial apoptosis pathway, which originates from the mitochondrial release of cytochrome *C* and the corresponding activation of caspase-9. Both of these pathways result in signaling cascades that converge on the activation of the executioner, caspase-3, which eventually results in apoptosis. However, there exists a third, less characterized pathway, which is essentially a second intrinsic pathway. This originates from the endoplasmic reticulum (ER) where there are damage sensors that link to apoptotic pathways and lead to the activation of caspase-12 and CHOP.^11^

Reticulons (RTNs) are membrane proteins located in the endoplasmic reticulum that have been recognized as molecular indicators of differentiation in neuronal and neuroendocrine cells and tissues.^12, 13, 14, 15, 16^ They are abundantly expressed in the brain and have important roles in trafficking of material from the ER to the Golgi apparatus, as well as in apoptosis.^17, 18, 19^ Proteins of the reticulon family consist of four members (RTN1-3 and RTN4/Nogo). The C-terminal regions of these proteins are highly conserved, while the N-terminal regions are different and specific for each reticulon.^20^ Notably, the reticulon family gene 1 (RTN1) was characterized by antibodies that stained a subset of neuroendocrine tissues and was formerly called neuroendocrine specific protein.^21^ The RTN1 family has three variants, namely, RTN1-A, RTN1-B and RTN1-C.^22^ It has been reported that RTN1-A is a novel mediator of chronic kidney disease progression that promotes renal injury through ER stress.^23^ In addition, human RTN1-A and RTN1-B, but not RTN1-C, were found to bind with AP50, a component of the AP-2 adaptor complex that mediates endocytosis.^24^ RTN1-C is expressed in neurons and neuroendocrine tissues and the expression is increased during differentiation in PC12 cells and neuroblastoma cell lines. Moreover, RTN1-C has been shown to interact with Bcl-xL, and this interaction has been shown to change the subcellular localization of Bcl-xL and reduce its anti-apoptotic activity.^25^ Although RTN1 has been shown to be involved in various cellular processes and disorders, such as cellular trafficking, autoimmune response, apoptosis, Alzheimer’s disease and regulation of ER stress and DNA damage-induced cell death,^18^ the mechanisms by which RTN1 exerts these effects in the context of cerebral ischemia/reperfusion injury have not yet been characterized.

In the present study, we reported that RTN1-C was specifically elevated both in the MCAO and the OGD/R models and that its increased expression induced mitochondrial and ER stress associated apoptosis in neural cells. Knockdown of RTN1-C *in vivo* attenuated cerebral ischemia and reperfusion injury, which was linked to decreased levels of mitochondrial and ER stress-associated apoptosis molecules. The data indicated, for the first time, that RTN1-C is a novel mediator of neuronal apoptosis that promotes cerebral ischemia/reperfusion injury.

## Results

### Upregulation of RTN1-C in the brains of cerebral ischemia/reperfusion

**Figure 1:**
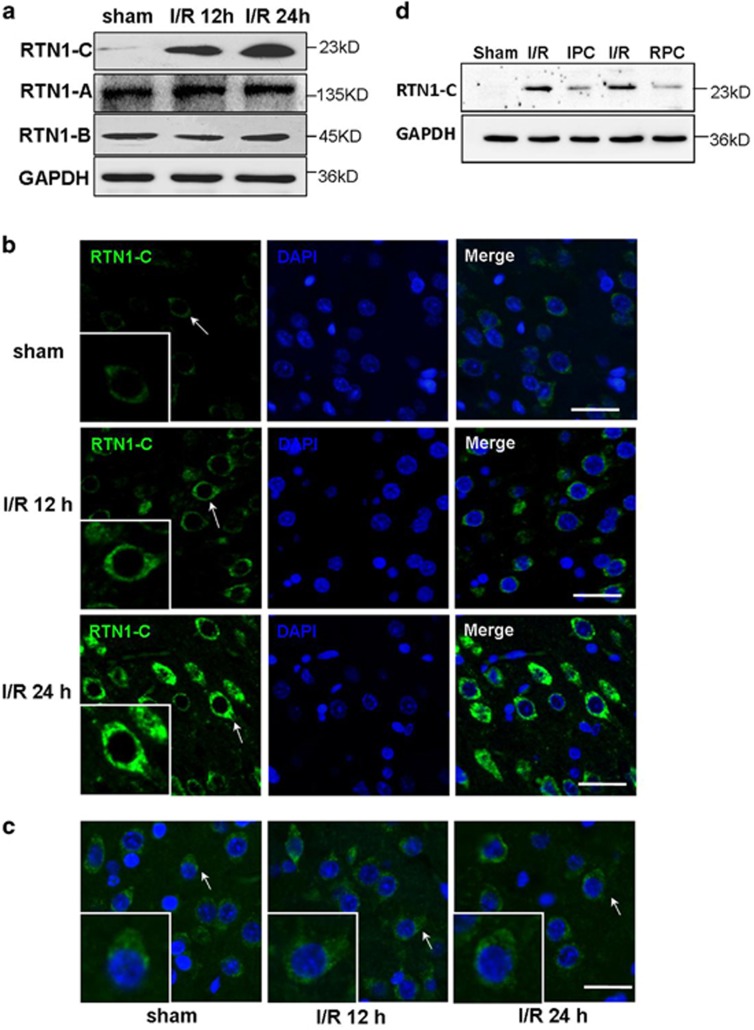
RTN1-C is upregulated during cerebral ischemia/reperfusion. Brain samples were collected from normal and ischemia/reperfusion (I/R) rat brain tissues. The male SD rats (*n*=3) were subjected to a middle cerebral artery occlusion (MCAO) for 2 h, followed by 12 or 24 h of reperfusion. (**a**) Representative western blot of three splice variants of RTN1 in the brain tissue samples from sham-operated and I/R rats. (**b**) Immunofluorescent staining of RTN1-C on brain sections from I/R rats. The upper panel is brain sections from sham-operated groups; the middle panel is the brain section from the group with MCAO for 2 h followed by 12 h of reperfusion; the lower panel is the brain section from the group with MCAO for 2 h followed by 24 h of reperfusion. Immunofluorescence was performed with anti-RTN1-C (green). DAPI was used to stain the nuclei (blue). The arrows highlight the RTN1-C expression. Scale bar=20 *μ*m. (**c**) Representative immunofluorescent staining of RTN1-A on the brain sections of I/R rats. Immunofluorescence was performed with anti-RTN1-C (green). DAPI was used to stain the nuclei (blue). Scale bar=20 *μ*m. (**d**) Representative western blot of RTN1-C in the brain tissue samples from I/R, ischemic postconditioning (IPC), Remifentanil postconditioning (RPC), and sham-operated rats

Previous studies suggested that RTN1 might be involved in the pathogenesis of human diseases, principally neurodegenerative disorders.^21, 23, 26^ To determine whether RTN1 is involved in cerebral ischemia/reperfusion (I/R) injury, we performed western blot to detect the expressions of the three splice variants of RTN1 in the brain tissue samples of MCAO rats. Among the three splice variants of RTN1, only RTN1-C staining was significantly upregulated by ischemia/reperfusion in rat brains (Figure 1a). To further confirm this result, we carried out RTN1 immunostaining on brain tissue samples of MCAO rats. The results showed that RTN1-C staining was stronger in the cortex of MCAO rats compared with sham-operated rats; particularly, it was more prominent after 2 h occlusion following 24 h reperfusion (Figure 1b). However, RTN1-A, another member of the RTN1 family, did not reveal any changes between MCAO and sham-operated rats (Figure 1c). Ischemic postconditioning is a promising strategy against cerebral ischemia/reperfusion injury owing to its clinical application.^27, 28^ Remifentanil is widely used in clinical anesthesia as an opioid analgesic. Previous studies have proposed that postconditioning with remifentanil protects global cerebral ischemia-induced spatial learning and memory deficit in rats via the inhibition of neuronal apoptosis.^29^ To explore the role of RTN1-C in cerebral ischemia/reperfusion injury, we further detected the expression of RTN1-C in the brain samples of MCAO-challenged rats during ischemic postconditioning (IPC) and remifentanil postconditioning (RPC). The rats were subjected to 2 h MCAO plus 5-min reperfusion plus 5-min reocclusion, then followed by 24 h reperfusion for IPC. For RPC, the rats were subjected to 2 h MCAO, followed by 24 h reperfusion, remifentanil (0.6 *μ*g/kg/min) was administered intravenously for 5 min at the onset of reperfusion for postconditioning.^29^ The results demonstrated that the levels of RTN1-C in the group with 2 h occlusion following 24 h reperfusion were significantly higher than those in the ischemic postconditioning or remifentanil-postconditioning groups (Figure 1d). The results thus suggested that the expression of RTN1-C was upregulated during cerebral ischemia/reperfusion and decreased during ischemic postconditioning. These observations supported the pivotal role of RTN1-C in cerebral ischemia/reperfusion injury.

### RTN1-C promotes apoptosis during cerebral ischemia/reperfusion

**Figure 2:**
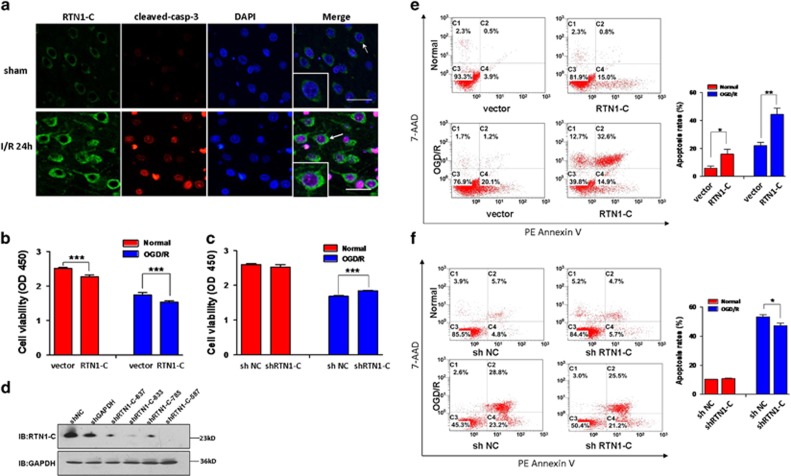
RTN1-C promotes apoptosis during cerebral ischemia/reperfusion. (**a**) Representative immunofluorescence photomicrography showing the expression of RTN1-C protein and cleaved-caspase-3 protein in sham-operated rats (the upper panels), or MCAO rats (the bottom panels). The expressions of RTN1-C (green) are shown by white arrows and magnification in merged panels. cleaved-caspase-3 (red) and the nuclei (DAPI, blue). Scale bar=20 *μ*m. (**b**) N2a cells were transfected with RTN1-C-GFP or GFP vector, then exposed to OGD for 3 h and reoxygenation for 4 h, or maintained under normal conditions. Cell viability was detected with CCK-8. *n*=5, ***P<0.001. (**c**) N2a cells were transfected with shRTN1-C or shNC (non-targeting shRNA), then exposed to OGD for 3 h and reoxygenation for 4 h, or normal conditions. Cell viability was detected with CCK-8. *n*=5, ***P<0.001. (**d**) The effectiveness of shRNA of RTN1-C was monitored by western blot. N2a cells were transfected with four different shRNA against RTN1-C (shRTN1-C- 637; shRTN1-C -833; shRTN1-C -785; shRTN1-C -587), shRNA against GAPDH and shNC (non-targeting shRNA) for 24 h, following by western blot analysis. (**e**) N2a cells were transfected with RTN1-C-GFP or GFP vector, then exposed to OGD for 3 h and reoxygenation for 4 h, or normal conditions. After OGD/R treatment, apoptosis in the cells was detected by flow cytometry with Annexin V-PE/7-AAD staining. *n*=3, *P<0.05; **P<0.01. (**f**) N2a cells were transfected with shRTN1-C or shNC (non-targeting shRNA), then exposed to OGD for 3 h and reoxygenation for 4 h, or normal conditions. After OGD/R treatment, apoptosis in the cells was detected by flow cytometry with Annexin V-PE/7-AAD staining. *n*=3, *P<0.05

To explore whether the expression of RTN1-C is relevant to I/R-induced apoptotic neural death, immunofluorescent staining for cleaved-caspase-3 (a specific effector for execution phases of apoptosis) was performed on the brain samples collected from MCAO and sham-operated rats. Consistently, we found that cerebral ischemia/reperfusion up-regulated the expression of RTN1-C, and the I/R brain tissues contained significantly more cleaved-caspase-3 positive cells compared to the sham-operated animals (Figure 2a). Next, we used overexpression and knockdown approaches to examine whether RTN1-C mediates cerebral ischemia/reperfusion injury. Before detecting the viability of neural cells, we confirmed the efficiency of the short hairpin RNA (shRNA) against RTN1-C by western blot analysis (Figure 2d). Cell Counting Kit-8 (CCK-8) assay showed that overexpression of RTN1-C significantly reduced cell viability of OGD/R-treated N2a cells (Figure 2b). Consistently, shRNA against RTN1-C also enhanced cell viability of the OGD/R-treated cells (Figure 2c). Furthermore, we confirmed that overexpression of RTN1-C enhanced the levels of apoptosis, while knockdown of RTN1-C attenuated it in the OGD/R-treated cells, as assessed by flow cytometry analysis using PE Annexin V (Figures 2e and f). These observations suggested that RTN1-C may promote apoptosis of neural cells during cerebral ischemia/reperfusion.

### RTN1-C induces ER stress-associated apoptosis during OGD/R

**Figure 3:**
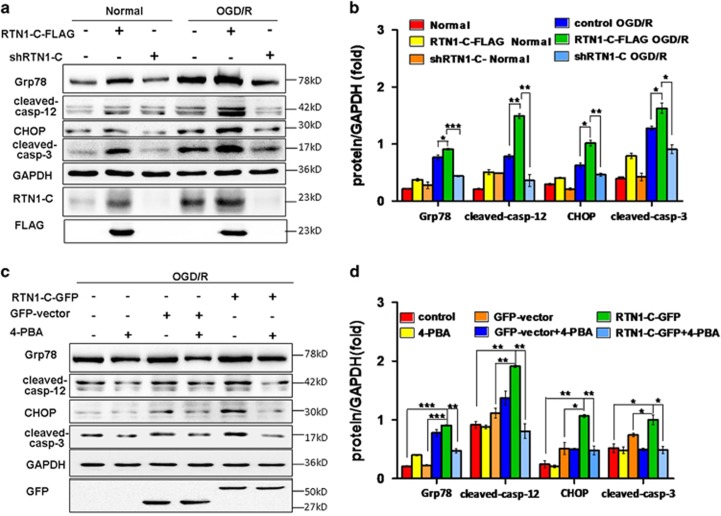
RTN1-C induces ER stress-associated apoptosis during OGD/R. (**a**) Representative western blot analysis of Grp78, cleaved-caspase-12, CHOP and cleaved-caspase-3 in N2a cells exposed to OGD/R treatment or normal conditions, after transfection with RTN1-C-FLAG or shRTN1-C. GAPDH was used as a loading control. (**b**) Histogram showing quantification of images in **a**. The results were normalized to GAPDH expression, and the quantitative data were expressed as the mean±S.D. from different assays (*n*=3). *P<0.05; **P<0.01; ***P<0.001. (**c**) Representative western blot analysis of Grp78, cleaved-caspase-12, CHOP and cleaved-caspase-3 in N2a cells transfected with RTN1-C-GFP or GFP vector in the absence or presence of 4-PBA for 6 h before OGD/R treatment. GAPDH was used as a loading control. (**d**) Histogram showing quantification of images in ***c**.* The results were normalized to GAPDH expression, and the quantitative data were expressed as the mean±S.D. from different assays (*n*=3). *P<0.05; **P<0.01; ***P<0.001

Many studies have suggested that ER stress is an initiator of cell death during hypoxia and ischemia/reperfusion. While the function of RTN1-C has been shown to be involved in the regulation of ER stress, the mechanisms by which RTN1-C exerts its effects during cerebral ischemia/reperfusion are not known. ER stress triggers the activation of ER-resident proteases, caspase-12, and CHOP which can participate in ER-associated apoptosis. To confirm the involvement of ER stress in OGD/R-induced apoptosis, the expression levels of the ER stress biomarker, Grp78, and the ER stress-associated apoptotic markers, CHOP, cleaved-caspase-12 and cleaved- caspase-3 were analyzed under conditions of the normal and OGD/R treatment in N2a cells. The results indicated that the overexpression of RTN1-C significantly increased the expressions of Grp78, cleaved-caspase-12, CHOP and cleaved-caspase-3 in OGD/R-treated cells compared to those in the case of RTN1-C knockdown in OGD/R-treated neural cells or cells under normal condition (Figures 3a and b). To further confirm RTN1-C involvement in ER-associated apoptosis, we transfected N2a cells with RTN1-C-GFP or GFP vectors and treated them with 4-PBA (ER stress inhibitor) for 6 h before OGD/R treatment. We found that the expressions of Grp78, cleaved-caspase-12 and cleaved-caspase-3 were significantly higher in the RTN1-C overexpression groups, while pretreatment with 4-PBA suppressed the expressions of these proteins (Figures 3c and d). These results suggested that RTN1-C induces neural apoptosis by mediating ER stress-dependent apoptosis during OGD/R.

### RTN1-C induces mitochondrial-associated apoptosis during OGD/R

**Figure 4:**
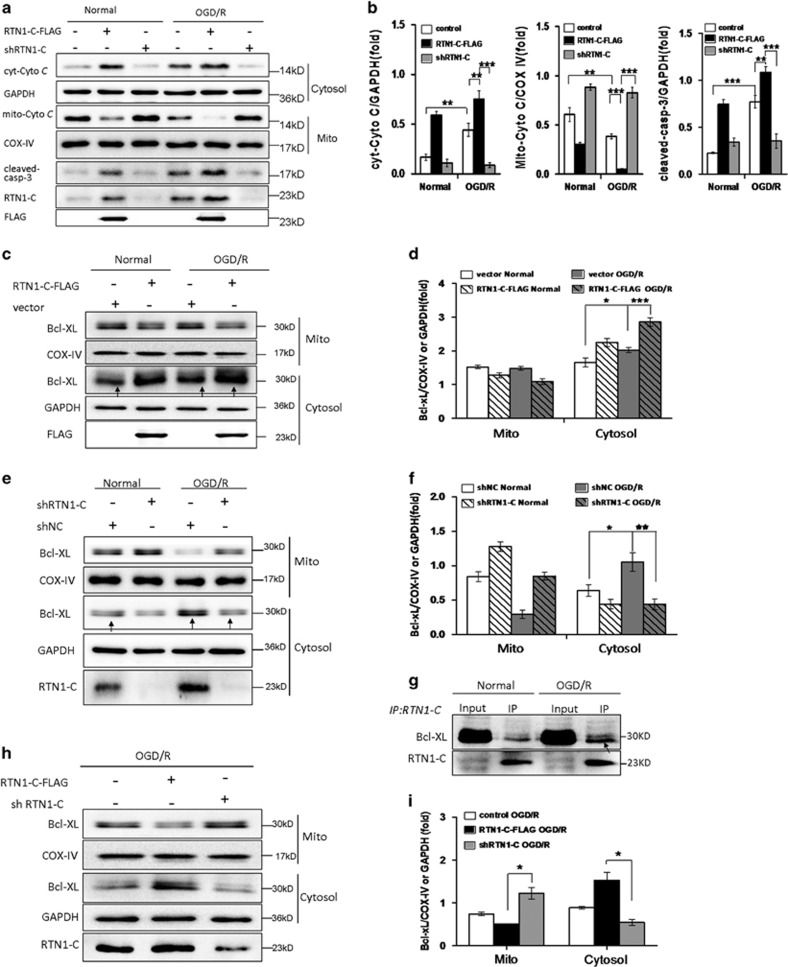
RTN1-C induces mitochondrial-associated apoptosis during OGD/R. (**a**) Representative western blot analysis of cytosolic cytochrome *C* (cyt-*Cyto C*), mitochondrial cytochrome *C* (mito-*Cyto C*) and cleaved-caspase-3 in N2a cells exposed to OGD/R treatment or normal condition, after transfection with RTN1-C-FLAG or shRTN1-C. GAPDH was used as a loading control. Fractionation experiment was performed before western blot. (**b**) Histograms showing quantification of images in **a**. The results were normalized to COX IV or GAPDH expression, and the quantitative data were expressed as the mean±S.D. on different assays (*n*=3). *P<0.05; **P<0.01; ***P<0.001. (**c**) Representative western blot analysis of mitochondrial and cytosolic Bcl-xL in N2a cells transfected with RTN1-C-FLAG or empty vector for 24 h, then the cells exposed to OGD/R treatment or normal condition. COX IV and GAPDH were used as the markers of mitochondria and cytosol, respectively. (**d**) Histogram showing quantification of images in ***c**.* The results were normalized to COX IV or GAPDH expression, and the quantitative data were expressed as the mean±S.D. on different assays (*n*=3). *P<0.05; **P<0.01; ***P<0.001. (**e**) Representative western blot analysis of mitochondrial and cytosolic Bcl-xL in N2a cells transfected with shRTN1-C or shNC for 24 h, then the cells exposed to OGD/R treatment or normal condition. COX IV and GAPDH were used as the markers of mitochondria and cytosol, respectively. (**f**) Histogram showing quantification of images in **e**. The results were normalized to COX IV or GAPDH expression, and the quantitative data were expressed as the mean±S.D. on different assays (*n*=3). *P<0.05; **P<0.01. (**g**) Immunoprecipitation of endogenous Bcl-xL and RTN1-C. N2a cells were treated with OGD/R and the lysates were incubated with anti-RTN1-C antibody. Bcl-xL and RTN1-C were detected by immunoblotting with anti-Bcl-xL and anti-RTN1-C antibody, respectively. (**h**) Representative western blot analysis of mitochondrial and cytosolic Bcl-xL in N2a cells exposed to OGD/R treatment after transfection with RTN1-C-FLAG or shRTN1-C. COX IV and GAPDH were used as the markers of mitochondria and cytosol, respectively. (**i**) Histogram showing quantification of images in **h**. The results were normalized to COX IV or GAPDH expression, and the quantitative data were expressed as the mean±S.D. on different assays (*n*=3). *P<0.05

Mitochondria are cellular organelles that play critical roles in the apoptotic pathway during ischemic injury.^30^ Previous studies have stated that cerebral ischemia/reperfusion generates free radicals, mainly released by the mitochondria, thereby resulting in oxidative stress in neurons. Overproduction of reactive oxygen species by mitochondria causes damage to proteins and lipids, which impairs mitochondrial function and leads to an increase in the mitochondrial membrane permeability. As a result, the permeabilized mitochondria release cytochrome *C*, activating caspases and leading to apoptotic cell death after ischemia/reperfusion.^31^ To ascertain whether RTN1-C contributes to I/R injury via mitochondria-dependent apoptosis, we analyzed the associated apoptotic markers, cytochrome *C* and cleaved-caspase-3. The results showed that cytosolic cytochrome *C* and cleaved-caspase-3 protein levels were greatly enhanced after OGD/R treatment and RTN1-C overexpression led to a dramatic increase of these proteins, which was relieved by RTN1-C knockdown (Figures 4a and b). As previously reported, RTN1-C interacts with Bcl-xL and translocates some of the Bcl-xL from the mitochondria to the ER, thus reducing its anti-apoptotic activity.^25^ To further evaluate the interaction between RTN1-C and Bcl-xL during OGD/R, the OGD/R-treated or normal treated cells were immunoprecipitated by the RTN1-C antibody. We found that endogenous RTN1-C interacted with Bcl-xL, and this interaction was enhanced in the OGD/R-treated cells compared to the normal treated cells (Figure 4g). To confirm whether RTN1-C changes the subcellular localization of Bcl-xL, we overexpressed RTN1-C-FLAG in the OGD/R-treated or no-treated cells. The mitochondrial and cytosolic fractions were isolated and the levels of Bcl-xL protein were detected. The results showed that RTN1-C overexpression greatly decreased the mitochondrial Bcl-xL and subsequently enhanced cytosolic Bcl-xL in the OGD/R-treated cells compared to the control or normal cells (Figures 4c and d). We further used knockdown approaches to confirm whether RTN1-C mediates Bcl-xL translocation. The result revealed that the cytosolic Bcl-xL was increased in the OGD/R-treated cells compared with the normal cells. However, when the OGD/R treated cells were transfected with shRTN1-C, the cytosolic Bcl-xL was significantly decreased, suggesting that RTN1-C promoted Bcl-xL to release from mitochondria during OGD/R (Figures 4e and f). In addition, we also confirmed that overexpression of RTN1-C significantly decreased the expression of mitochondrial Bcl-xL in the OGD/R-treated cells compared to RTN1-C knockdown in the same cells (Figures 4h and i). Collectively, these results suggested that Bcl-xL, at least partly, changed its subcellular localization from the mitochondria to the ER by binding with RTN1-C, thereby reducing its anti-apoptotic activity during OGD/R. Therefore, we concluded that RTN1-C induced mitochondrial-associated apoptosis during OGD/R by affecting Bcl-xL subcellular localization.

### Knockdown of RTN1-C reduced apoptosis during cerebral ischemia/reperfusion

**Figure 5:**
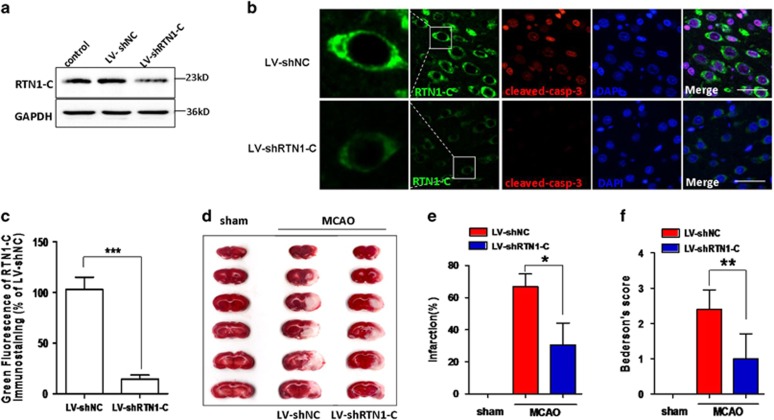
RTN1-C downregulation using its specific shRNA reduced neural apoptosis in MCAO rats. (**a**) Rats were given intracerebroventricular injections with LV-shNC or LV-shRTN1-C for 7 days, then the rats were subjected to a middle cerebral artery occlusion (MCAO) for 2 h, followed by 12 or 24 h of reperfusion. The efficiency of LV-shRTN1-C in the brain tissues was detected by western blot. (**b**) Representative immunofluorescence staining of cleaved-caspase-3 in MCAO rats after given intracerebroventricular injections with LV-shNC or LV-shRTN1-C for 7 days. Cleaved-caspase-3 (red), RTN1-C (green) and DAPI (blue), Scale bar=20 *μ*m. (**c**) Quantification of green fluorescence intensity of RTN1-C immunostaining in (**b**). Data are expressed as mean±S.D. *n*=5, ***P<0.001. (**d**) Representative TTC stains of 6 corresponding coronal brain sections. Ischemic infarctions appear white. (**e**) Brain infarct volumes were quantified. *n*=3, *P<0.05. (**f**) Neurological Bederson score assessed at day 1 after reperfusion in MCAO rats. *n*=5, **P<0.01

We have shown that RTN1-C induced apoptosis in OGD/R neural cells (Figures 3 and 4). To study whether RTN1-C downregulation could protect neurons against apoptosis in I/R rats and promote their survival, we employed lentivirus-based shRNA to down-regulate the RTN1-C expression. To confirm whether the lentivirus induces RTN1-C knockdown in brains, RTN1-C expression was determined by western blot, and the result confirmed the efficiency of the RTN1-C-specific shRNA lentivirus (LV-shRTN1-C), compared to the non-targeting shRNA (LV-shNC) and the control brain without any injection (Figure 5a). We also examined the effect of RTN1-C knockdown on neural apoptosis *in vivo*. Immunofluorescence staining for cleaved-caspase-3 showed that RTN1-C downregulation greatly decreased neural apoptosis compared to LV-shNC in the MCAO groups (Figures 5b and c). To further determine the involvement of RTN1-C in I/R injury, TTC staining was used to detect the therapeutic effect of LV-shRTN1-C on focal cerebral ischemia. No infarct area was observed in the sham group, while significant infarction was observed at 24 h after reperfusion. Under the injection with LV-shRTN1-C, the infarct volume significantly decreased compared with that with LV-shNC in the MCAO group (Figure 5d). The mean infarct volumes were 30.6±13.6 mm^3^ versus 70.0±7.8 mm^3^ in the LV-shRTN1-C and the LV-shNC groups, respectively (Figure 5e; *P*<0.05). We then used the Bederson score to assesses neurological function and study the effects of RTN1-C in MCAO rats. The results showed that LV-shRTN1-C treated rats exhibited significant improvement in neurological functions compared to LV-shNC groups (Figure 5f; *P*<0.01). Altogether, our findings demonstrated that RTN1-C could be important for cerebral ischemia/reperfusion injury and RTN1-C downregulation could protect the brain from ischemic damage and restore neurologic function impaired by ischemia.

## Discussion

Previous studies have demonstrated that RTN1-C can interact with glucosylceramide synthase (GCS) at the Golgi/ER interface. It has been shown to exert a cancer-specific proapoptotic function by affecting the response to fenretinide-induced apoptosis through a p53-independent.^21, 32^ It has also been reported that the increase in the level of RTN1-C protein leads to ER stress-induced cell death and significantly sensitizes cells to different ER stress inducers, whereas the reduction in RTN1-C protein levels dampens the response to ER stressors. In addition, RTN1-C also induces apoptosis by releasing cytochrome *C* from the mitochondria and thereby facilitating the activation of caspase-3.^22^ Recent research has provided evidence to support that RTN1-C is involved in the regulation of ER-mitochondria cross-talk by enabling contact between the two organelles.^33^ These studies indicate that RTN1-C mediates the ER-mitochondria cross-talk and induces apoptosis related to ER stress and mitochondria.

Identification of new biomarkers and drug targets for cerebral ischemia is required for the development of more effective therapies. Overwhelming evidence has suggested that in addition to necrosis, apoptosis also contributes significantly to cell death following cerebral ischemia/reperfusion.^9, 10^ Recent studies have shown that ER stress is a critical component of the pro-apoptotic signaling pathway and in the sensing of brain injury after I/R.^34, 35, 36^ During the ischemic process, energy depletion and disruption in calcium homeostasis may trigger the accumulation of misfolded proteins in the ER lumen, thereby activating the unfolded protein response (UPR). UPR activation can help reestablish homeostasis and normalize ER function, but if the injury is excessive, it can also lead to cell death.^37^ ER stress also activates caspase-12, a member of the ICE (interleukin-1*β* converting enzyme) subfamily of caspases, which is localized to the ER and expressed at moderate levels in the brain. CHOP is a transcription factor whose expression is upregulated during ER stress and it also participates in ER-mediated apoptosis.^38^ In this study, we demonstrated that RTN1-C, a member of the RTN family proteins, was upregulated following ischemia/reperfusion in rat brains and OGD/R-treated neural cells. RTN1-C exacerbated cell vulnerability to I/R injury and promoted apoptosis. In addition, our results here demonstrated that RTN1-C overexpression induced the expression of Grp78, cleaved-caspase-12 and CHOP during OGD/R. Furthermore, we have found that 4-PBA, which is a potent ER stress inhibitor, markedly reduced the RTN1-C-induced upregulation of Grp78, cleaved-caspase-12 and CHOP markers in OGD/R cells. These findings suggest that RTN1-C is one of the crucial players in ER stress-associated apoptosis during the I/R pathologic condition.

During the pathogenesis of cerebral ischemia/reperfusion injury, mitochondria play a critical role in promoting apoptosis via the reduction of mitochondrial membrane potential, the depletion of ATP synthesis, and the induction of increased membrane permeability.^39, 40, 41^ As a result, cytochrome *C* molecules are released from the mitochondria into the cytosol, to form a complex with Apaf-1, which binds to caspase-9 and activates it. This cascade further activates terminal caspases such as caspase-3, which in turn lead to cell death.^42, 43, 44^ The Bcl-2 family includes proteins that contain Bcl-2 homology domains. Some Bcl-2-like proteins (for example, Bcl-2 and Bcl-xL) have pro-survival functions, whereas others (such as BAX and BAK) are mainly pro-apoptotic. Bcl-2 and Bcl-xL are can stably insert themselves into the outer mitochondrial membrane and inhibit mitochondrial membrane potential.^45^ RTN1-C can interact with Bcl-xL, without affecting its levels, but change its subcellular localization from the mitochondria to the ER. Thus, RTN1-C can reduce the anti-apoptotic activity of Bcl-xL by preventing its effect on the mitochondria.^25, 46, 47^ Our study here showed that due to the binding with RTN1-C, Bcl-xL changed its subcellular localization from the mitochondria to ER, thereby reducing its anti-apoptotic activity during OGD/R.

Taken together, our data indicated that RTN1-C expression is highly upregulated during the progress of cerebral ischemia/reperfusion and that RTN1-C may be a key molecule in mediating ER stress and mitochondria-associated apoptosis in neural cells, thus contributing to the pathogenesis of cerebral ischemia/reperfusion injury. Importantly, we showed that the inhibition of RTN1-C expression attenuates ischemic damage and partly restores the neurologic functions impaired by ischemia/reperfusion, therefore suggesting that it may be a novel therapeutic target for stroke.

## Materials and methods

### Ethics Statement

Ethical permission was obtained from the Institutional Review Board of the Institute of Biomedicine at Anhui Medical University (permit number AMU27–0910202), which records and regulates all research activities in the school. The Institutional Review Board of the Anhui Medical University approved both animals and humans protocols. The approval from the Institutional Review Board includes the permission of using mouse under euthanasia, and all the experimental procedures were carried out in strict accordance with the recommendations in the Guide for the Care and Use of Laboratory Animals of the National Institutes of Health.

### Reagents and plasmids

The full-length human RTN1-C was cloned in our previous study^48^ and was inserted into pEGFP-C2 (BD Biosciences, San Diego,CA, USA) or p3FLAG-myc-CMV (Sigma, St. Louis, MO, USA). The oligonucleotides for short hairpin RNA (shRNA) were Sequence-Selector and synthesized from GenePharma (Shanghai, China). The oligonucleotides for shRNA were synthesized as follows: shRTN1-C-587: CACCGGAGCTTGAGATCACCCTTTCTTCAA GAGAGAAAGGGTGATCTCAAGCTCCTTTTTTG; shRTN1-C-637: CACCGCCTGCAGTTCTACGTGAACATTCAAGAGATGTTCACGTAGAACTGCAGGCTTTTTTG;shRTN1-C-785: CACCGGCTGTGGTTTCAATGTTTACTTCAAGAGAGTAAACATTGAAACCCACAGCCTTTTTTG; shRTN1-C-833: -CACCGGCACAGATTGACCAATATCTTTAAGA-GAAGATATTGGTCAATCTGTGCCTTTTTTG, were inserted into *BbsI* and *BamHI* sites of the pGPU6/Neo to generate pGPU6/Neo/RTN1-C. pGPU6/Neo/NC containing a scrambled sequence (NC) of shRNA was used as the negative control. pGPU6/Neo/GAPDH containing shRNA sequence that targets glyceraldehyde 3-phosphate dehydrogenase (GAPDH) was used as positive control. Lentiviral vectors were produced as previously described.^49^ The titres of the vectors in this study were 5 × 10^8^ TU/ml. 4-PBA (P21005, Sigma, USA). Two kinds of RTN1-C antibodies were purchased from Santa Cruz (Dallas, TX, USA) (sc-71982, Santa Cruz, USA) and Proteintech (15048-1-AP, Proteintech, China). RTN1-B (sc-53008, Santa Cruz, USA) and GAPDH (60004-1-lg, Proteintech, China), CHOP (sc-793, Santa Cruz, USA) were used. Antibodies against caspase-12 (#2202), cleaved-caspase-3 (#9664), GFP (#2956), BCL-xL (#2764), cytochrome *C* (#4272) and COX IV (#4850) were purchased from Cell Signaling Technology (CST, Danvers, MA, USA). The secondary antibodies, HRP-conjugated anti-rabbit and anti-mouse IgG, were purchased from Proteintech.

### Animals

Male Sprague-Dawley (SD) rats (grade SPF, weighing 240–260 g) were purchased from the Anhui Experimental Animal Center (Hefei, China). The rats were housed in an environment with standard lighting conditions (12 h light/dark cycle), controlled temperature (20-25 °C) and humidity (40-60%), and with freely accessible food and water. All experiments were carried out in accordance with the European Union directive (EEC Council 86/609; D.P.R. 116/92). All the experiments described in this study were approved by the Anhui Medical University Animal Care and Use Committee.

### Middle cerebral artery occlusion

The animal study was approved by the Animal Care and Use Committee of Anhui Medical University. All SD rats were treated according to the Guide for the Care and Use of Laboratory Animals. Male SD rats were obtained and bred as described previously.^50^ The focal ischemia models were set up by middle cerebral artery occlusion as described previously.^50, 51^ Briefly, the rats were anesthetized and the right common carotid artery was exposed to allow for the insertion of a nylon filament (0.235 mm in diameter) to the end of the internal carotid artery to block the origin of the right middle cerebral artery. After 2 h or 4 h of the occlusion, the nylon filament was withdrawn to enable reperfusion for 12 h or 24 h. Sham-operated mice underwent the same surgical procedure with the exception that their origin of the middle cerebral artery was not occluded. All animals were operated on by the same operator under the same conditions to reduce infarct variability. The operation time per animal did not exceed 15 min. The rats were sacrificed under deep anesthesia.

### Intracerebroventricular injection

The rats were anesthetized as described above and placed prostrated on a stereotaxic instrument (Stoelting Co, USA). A midline scalp incision was made to expose the skull and bregma. Five microliters of the lentivirus or vehicle was injected into the right lateral ventricle at a rate of 0.5 *μ*l/min with the coordinates of 1.5 mm lateral to the midline,^52, 53^ 1.0 mm posterior to the bregma, and 4.5 mm deep.^54^ The needle was held in place for 5 min after injection to prevent reflux. The rats were subjected to MCAO at 7 days after injection.

### Infarct volume assessment

The infarct volume was assessed by 2,3,5-triphenyltetrazolium chloride (TTC, Sigma-Aldrich, USA) staining. The rats were decapitated immediately after reperfusion and the brains were removed and frozen for 5 min at −80 °C. Then, the brains were sliced into 2.0-mm-thick sections and incubated in a 2% TTC solution for 30 min at 37 °C. Images were then captured using a digital scanner (HP Scanjet 200, USA). Infarction volumes were determined from the unstained areas, measured using an image analysis software (ImageJ). The infarct volume was calculated as a percentage using the following equation: infarct percentage=((contralateral hemisphere area−non-infarcted region in the ipsilateral hemisphere)/contralateral hemisphere area) × 100%

### Immunofluorescent staining

The harvested brains were fixed in 4% paraformaldehyde for 48 h and embedded in paraffin. Then, 5.0-*μ*m-thick brain sections were rehydrated and rinsed in PBS. After antigen retrieval, the sections were permeabilized and blocked with PBS containing 0.5% Triton X-100 and 5% goat serum for 1 h at 37 °C, and then incubated with primary antibody at 4 °C overnight. After washing, the sections were incubated with FITC-conjugated goat anti-rabbit IgG (Abcam, Cambridge, MA, USA) for 1 h at 37 °C. DAPI dye was used for DNA visualization. The images were captured using a fluorescence microscope (Olympus, Japan).

### Neurological function test

Neurological function was evaluated using a modification of the Bederson neurological scale.^55, 56, 57^ Neurological scores were recorded as follows: 0, no neurological deficit; 1, failure to fully extend left forepaw or flexion of torso and contralateral forelimb when mouse was lifted by the tail; 2, reduced resistance to lateral push or circling to the contralateral side when mouse was held by the tail on a flat surface, but normal posture at rest; 3, spontaneous circling to left; 4, the absence of spontaneous movement or unconsciousness.

### Cell cultures

The mouse neuroblastoma cells, N2a (ATCC), were cultured in DMEM (HyClone, USA) supplemented with 10% FBS and 1% penicillin/streptomycin in a humidified incubator with 5% CO_2_.

### Oxygen-glucose deprivation and reoxygenation (OGD/R) injury

Oxygen-glucose deprivation and reoxygenation was performed as a model of *in vitro* I/R. First, the medium was replaced with glucose-free DMEM (Gibco, USA) and the plates were placed in an anaerobic chamber containing 95% N_2_ and 5% CO_2_ at 37 °C for 3 h. Then, the N2a cells were returned to normal medium and incubated under normal conditions to terminate the OGD and start reperfusion.

### Cell viability assay

Cell viability was evaluated using a Cell Counting Kit-8 (Dojindo, Japan) according to the manufacturer’s instructions. N2a cells were plated into 96-well plates and 10 *μ*l CCK-8 solution was added to each well for 2 h at 37 °C. The absorbance was measured at 450 nm to determine cell viability.

### Detection of apoptosis

Apoptosis in the N2a cells was determined with the Annexin V-PE/7-AAD kit (BD, USA), according to the manufacturer’s instructions. The N2a cells were collected and washed twice with cold PBS, then incubated in 1 × binding buffer (10 mM HEPES/NaOH (PH 7.4), 0.14 M NaCl, and 2.5 mM CaCl_2_) containing PE Annexin V and 7-AAD for 15 min. Then, the stained cells were analyzed using a flow cytometer (Beckman, Boulevard Brea, CA, USA) within 1 h. Cells that stained positive for PE Annexin V were considered to be undergoing apoptosis.

### Western blot analysis

Cells or brain tissues were lysed in RIPA lysis buffer (Beyotime, China) supplemented with a protease inhibitor 1 mM phenylmethylsulfonyl fluoride (PMSF, Sigma, USA). The lysates were separated by SDS-PAGE and transferred onto nitrocellulose membranes (Millipore, Billerica, MA, USA). The membranes were blocked with 5% skim milk in TBST for 1 h and incubated with primary antibodies overnight at 4 °C. After washing with TBST, the membranes were incubated with corresponding peroxidase-conjugated secondary antibodies for 1 h, and then washed and developed using the ECL chemiluminescent detection system.

### Immunoprecipitation assay

Whole cell lysates were prepared 24 h after transient transfection in a lysis buffer containing 20 mM Tris (pH 8.0), 138 mM NaCl, 10% glycerol, 1% Nonidet P-40, 10 mM NaF, 2 mM NaVO_4_, 1 mM pyrophosphoric acid and Complete protease inhibitors (Roche Applied Science, Indianapolis, USA). The supernatants were collected and incubated with Protein A/G plus-agarose (Pierce) and relevant antibodies for 2 h at 4 °C. The bound proteins were then eluted and subjected to western blot analysis.

### Mitochondria isolation

All steps were carried out in accordance with the instructions of the Cell Mitochondria Isolation Kit (Beyotime, China). First, 5 × 10^7^ cells were prepared and washed with cold PBS and centrifuged at 4 °C 600 g for 5 min. Second, the mitochondria isolation reagent with 1 mM PMSF was mixed with the cells for 15 min, and the mixture was ground 50 times using a homogenizer. The cell homogenates were centrifuged at 4 °C 600 g for 10 min. The supernatants were transferred to new tubes and centrifuged at 4 °C 11000 g for 10 min. The precipitate containing mitochondria was lysed with mitochondria lysis buffer for western blot.

### Statistical analysis

We used SPSS statistical software (version 16.0, SPSS, Chicago, IL, USA). The data are expressed as the mean±S.D. Differences between the data were tested for statistical significance using *t*-test. *P*<0.05 was considered statistically significant.

## Publisher’s Note

Springer Nature remains neutral with regard to jurisdictional claims in published maps and institutional affiliations.
